# A latent diffusion approach to visual attribution in medical imaging

**DOI:** 10.1038/s41598-024-81646-x

**Published:** 2025-01-06

**Authors:** Ammar Adeel Siddiqui, Santosh Tirunagari, Tehseen Zia, David Windridge

**Affiliations:** 1 Middlesex University, London, UK; 2 COMSATS University, Islamabad, Pakistan

**Keywords:** Visual Attribution, Explainable AI, Diffusion models, Medical imaging, Computational biology and bioinformatics, Computer science, Health care

## Abstract

Visual attribution in medical imaging seeks to make evident the *diagnostically-relevant* components of a medical image, in contrast to the more common detection of diseased tissue deployed in standard machine vision pipelines (which are less straightforwardly interpretable/explainable to clinicians). We here present a novel generative visual attribution technique, one that leverages latent diffusion models in combination with domain-specific large language models, in order to generate *normal counterparts* of abnormal images. The discrepancy between the two hence gives rise to a mapping indicating the diagnostically-relevant image components. To achieve this, we deploy image priors in conjunction with appropriate conditioning mechanisms in order to control the image generative process, including natural language text prompts acquired from medical science and applied radiology. We perform experiments and quantitatively evaluate our results on the COVID-19 Radiography Database containing labelled chest X-rays with differing pathologies via the Frechet Inception Distance (FID), Structural Similarity (SSIM) and Multi Scale Structural Similarity Metric (MS-SSIM) metrics obtained between real and generated images. The resulting system also exhibits a range of latent capabilities including *zero-shot localized disease induction*, which are evaluated with real examples from the cheXpert dataset.

## Introduction

Medical imaging has become increasingly important in modern medical settings for patient stratification, assessing disease progression, evaluating treatment response, and grading disease severity^1^. However, medical image diagnosis tends to involve far more than simple disease detection. Visual Attribution (VA) is the detection, identification and visualization of *evidence*of a particular class or category of images^2^. It is a specific part of explainability of learned models i.e using visualization techniques to investigate the decisions made by a model, and attribute the decisions to distinct parts of an image. This opens the model to interpretation, a key aspect of XAI (Explainable AI) machine learning research, especially in relation to deep learning models^3^.

As it manifests, in medical imaging, VA is the process of educing evidence for medical conditions in relation to different parts of an image, such as pathological, psychological or disease-related effects^4–7^. As such, VA differs from the straightforward detection or segmentation of pathological regions in standard medical machine vision. These detected or segmented parts of the image are thus crucial biomarkers, and may serve as additional diagnostic and prognostic evidence^8^. Such models base their decisions on locally or globally perceived evidence components, and it is thus in these terms that the VA aspects of the models must be visually and semantically interpretable^9^. In clinical practice, these findings may then be used to diagnose and select treatment options, which may be surgical intervention, prescription of drugs etc. Interpretability is also key for scientific understanding of the system as a whole, and VA knowledge may thus sit on top of the explicit output of the model (for example, VA-based delineation of those regions *affected*by a tumor, typically extending significantly beyond the segmented tumor region itself). VA knowledge factors may also relate to the safety of the application, or to the ethics and a priori biases of the data, highlighting incomplete or mismatched objectives being optimized by the model^10^.

A lack of interpretability of one or more of these examples may lead to complete or partial system failure, the model failing to achieve some aspect of the complex targets provided by the user/clinician, or optimization of an objective different to that intended. Model explainability is hence of critical interest in the medical imaging domain, having been identified as crucial to increasing the trust of medical professionals in the automated diagnostic domain^1^. Visual attribution consequently provides a way to increase the confidence between the system, patient and clinician, leading to fewer misinformed results^11^. It may also serve to decrease cognitive load on the clinicians and medical practitioners via automated localization and segmentation of areas of interest^12,13^. However, it is important to consider the specific requirements and safety-criticalities of the application when developing a VA model (methods that directly manipulate images in the pixel space typically have to gain the acceptance of diagnosticians as part of their work process^14^), and use-case flexible human-in-the-loop models are therefore to be preferred in the general case.

### Generative visual attribution

The most recent techniques in visual attribution involve variants of deep neural networks (DNNs), which tackle the problem in different ways, though typically centred on classification or segmentation^15,16^. The need for VA is especially acute for DNNs in a clinical setting due to their intrinsic high complexity and low interpretability, often termed ‘black boxes’^17,18^. However, DNNs, uniquely amongst machine learning VA approaches have the capacity to act in a *generative* manner. They hence have the capacity to mimic the actual clinical practice of a radiologist or practitioner, typically trained via the *difference*between healthy and non-healthy disease manifestations. As a result, the diagnosis of a condition or disease may be implicitly explained in terms of abnormalities of non-healthy tissue in relation to a hypothetical healthy version of the same tissue^19^.

Generative DNN-based machine learning therefore leads to the state-of-the-art strategy of *generative visual attribution* (developed in part by the authors) that leverages generative methods for counterfactual normal generation, in which abnormal images are translated into their *normal counterparts*for observation by a clinician. These methods hence perform visual attribution map generation via heatmaps taking the difference between the observed image of a patient and its healthy counterfactual^19–21^.

Previously, such techniques have used a specific DNN generative mechanism, *Generative Adversarial Networks* or *GANs*to carry out this mapping (cf the techniques ANT-GAN^19^and VANT-GAN^20^). This attribution process exploits the underlying properties of GANs to directly model the differences present between the normal and abnormal clinical images, as well as capture the complete structure of the individual classes in a learned latent representation. GANs in general have the advantage of requiring relatively fewer abnormal examples^22^than standard supervised learning while still capturing underlying features of the surrounding areas of the higher density information regions. (Examples of these overlooked regions might be micro tumors in other parts of an organ that may not, in themselves, have a highly significant effect on the supervised decision boundary^2^; it has been shown, especially for medical imaging DNNs, that such models typically disregard a significant fraction of these regions, which are essentially background evidence in relation to the underlying pathological condition^23^).

However, GANs, while powerful, have faults that have led to the very recent development of a new state-of-the-art generative mechanism: *visual diffusion*. Diffusion models are typically able to operate at higher resolutions and image qualities than GANs. They are also superior to GANs in not suffering from ‘mode collapse’ arising from the adversarial process of distinguishing real from generated images reaching a convergence (Nash equilibrium) in which critical image classes are omitted^24^. Diffusion models have been used for counterfactual generation as Diff-SCM^21^, and similar^19,25,26^.

Latent Diffusion models, however are not without limitations, and inevitably exhibit systemic biases in common with other generative deep learning architectures. One such limitation of relevance in medical settings is that of *mode interpolation*^27^- a particular generative hallucination that combines similar modes, giving rise to artefacts that are over-determined by the training data. In both GANs and Diffusion models this issue can be resolved by the addition of training data. More problematically in the medical setting, a hallucination phenomenon commonly observed in GANs is that of circular artefacting^28^, which acts to degrade overall image quality via the hallucination of ’blobs’. This has the potential to be exceptionally detrimental for therapeutic applications, given that objects of interest are often manifested as localised circular features such as clots, tumors, blood cells etc., which the artefacts have the strong potential to mimic. Fortunately, these artefacts are much rarer in the latent diffusion domain, and this is a key motivation underlying the current study. Failure modes that are more specific to diffusion models include training instabilities^29^, and memorization^30^. However, these would appear to be less intrinsically problematic in a medical context.

In this work, we shall thus propose to extend the VANT-GAN technique by deploying visual diffusion for counterpart normal generation. Our approach hence uses counterfactual generation with diffusion models directed at visual attribution in the medical imaging domain in a manner that builds on the conceptual foundations of generative visual attribution laid out in VANT-GAN^20^. In doing so, we will aim to increase the interpretability of the model by using multi-modal (text and image) inputs. We hence leverage prior control and conditioning techniques to reliably steer the mapping process in an interpretable manner utilising text prompts and control images. We achieve this by training domain-specific language and vision models on relevant medical imaging data allowing the generation of visual attribution maps for specific medical conditions, which can be quantitatively measured using relevant metrics in the domain.

The proposed architecture thus builds on extant methodology in a number of key computer-vision areas, in particular conditional image generation/image translation, saliency mapping and counterfactual medical image generation, including the deployment of multiple and conditional decoders^31^to generate saliency maps for reconstruction, joint-training of generative and inference components^32^, multi-stage bootstrapped training via an encoder^33^, use of frozen generative networks, and the use of latent representations of disease images^34^. The architectural model chosen for this study, specifically the conditional latent diffusion pipeline, thus combines and enhances methodology from the aforementioned approaches in an efficient package (in in terms of the number components used) while addressing the deficiencies of the VANT-GAN approach. We believe that the overall improvement in image quality, avoidance of mode collapse, readily connectable pretrained components via cross attention using e.g. CLIP and BERT based models, and quick end-to-end joint fine tuning makes the pipeline and ideal choice for deployability in the XAI domain, including that of the medical domain.

As well as improving reliability, trustworthiness and utility with respect to previously applied techniques of generative visual attribution, the approach of utilizing diffusion models in combination with domain-adapted large language models with enhanced controllability and conditioning potentially also opens horizons to applications such as *post-surgery simulation of ageing, disease* etc by leveraging natural language instructions, as well as a host of additional ‘zero-shot’ latent use-case capabilities.

#### Diffusion generative models

Diffusion models consist of an autoencoder, which encodes the image into a latent space, and a diffusion process in which stochastic perturbations are performed incrementally in the latent space, such that a DNN can learn the reverse denoising process capable of transforming random noise images into images from the trained domain (a process which may be guided by a suitable language model to introduce linguistic priors in the image generation). Depending on the autoencoder, the images generated by diffusion models are typically of relatively high resolution (compared with GANs) and the textual conditioning may include a wide range of textual encoders trained on specific domains, e.g. in the medical domain BioBERT^35^, RadBERT^36^and PubmedCLIP^37^. Such language encoders can hence be used to condition the generation in a much more flexible way than other generative models, in particular GANs.

Other approaches use the metadata in the datasets to help learn models that take into account age, gender, intracranial and ventricular volume etc in parallel with image conditioning such as RoentGen^38^and LDM+DDIM^39^ for synthetic image generation. This meta-information can then be used to measure correlation among real images.

This ability to guide diffusion models via external semantic model make them potentially very powerful and relevant to visual attribution, especially in the medical imaging domain.

### Proposed methodological approach

The current research builds upon a particular conception of generative visual attribution set out in^20^ in the context of GAN generative models. In particular, it seeks to build on the notion of *counterpart normal generation*, but enriched via the use of visual diffusion and large language models.

We thus leverage domain-adapted language components combined with conditional generation to modify the latent diffusion in a manner suited to medical VA. The approach hence combines domain-adapted large language and vision models to enable broad medical understanding to be brought to bear on the problem of counterpart normal generation, enabling generative visual attribution useful to understanding and pinpointing visual evidence in the form of generated counterfactuals and visual maps. Additionally, the representative power of the domain adapted large language model alongside the image-domain representation of the vision model ensures that medical image concepts are grounded in medical language, such that counterfactual generation may be prompted via complex (natural language) text prompts including, potentially, location and intensity of disease or condition, or else constrained to the specific organs within a medical scan. Note that the vision model is not directly trained on such morphological concepts beforehand (e.g. the concept of an organ or the boundaries of an organ), yet is able to extrapolate from the combined multimodal knowledge using the data from the language and visual domain to discover these concepts latently.

Lastly, the model proposed shows zero-shot generation capabilities on disease concepts that are out of the training data distribution, but which also appear qualitatively valid in the generated counterfactuals. This is presumably the result of exploiting the different extrapolate capabilities of the respective vision and language models in a synergistic manner. The model thus latently encompasses the ‘rules of biology’ in generating counterfactuals, e.g not generating extra lung scar tissue where it could not exist, outside of the chest cavity, irrespective of the language prompt.

This strengthens our argument for using latent diffusion models for visual attribution, since no direct perturbations are made in pixel space and neither is the model trained on synthetic data. We also need only use a dataset with a modest amount of images and basic one-word labels, relying on the text encoder (pretrained on domain-specific data, e.g. radiology reports) to supply additional linguistic concept relations.

The contributions of the study are as follows:

1. We illustrate the use of the visual diffusion pipeline for jointly fine-tuning the combination of a domain-adapted text encoder and a vision encoder with a modest amount of real medical scans and text prompts for conditional scan generation (we thus eliminate the need for synthetic data).
2. We generate visually valid counterfactuals (non-healthy to healthy and vice versa) with minimal perturbations to the original real image guided by text prompts that employ complex natural language medical imaging concepts.
3. We explore the interpolation of knowledge in the text and vision domains using the composite text/vision models, evaluating the validity of the interpolations in the respective language and vision domains via their reflection into the other.
4. Using the generated counterfactuals, we generate visual maps by subtracting the generated counterfactual from the original image for visual attribution in the medical imaging domain, thereby enhancing diagnostic explainability in the manner of VANT-GAN (motivating the use of these models in safety-critical diagnostic applications in which visual explanation is critical for highlighting different areas of interest).
5. We show zero-shot generation capabilities in the visual domain for inducing diseases in healthy or non-healthy scans prompted by complex text prompts including medical imaging concepts using the text encoder. We perform and ablation study, eliminating components of the pipeline to investigate the individual and collective contribution of the text encoder and image priors aspects of the pipeline.
6. Finally, we indicate the potential for future studies using such a combination of vision and language concepts for visual attribution using conditional generation.

## Related work in generative visual attribution

### Generation of activation maps

Generative visual attribution includes a variety of classes of approach, each of which tackle the explainability problem in different ways. The particular class emphasised here, exemplified in a^2,19^and^20^, seek to generate complete or partial counterfactuals of the abnormal (i.e. diseased) image, and generate implicitly or explicitly a discrepancy map between the two. These maps are then visualized to highlight the attributing parts of the normal or abnormal image.

The ANT-GAN^19^approach hence leverages GANs to generate normal or healthy-looking images from abnormal or unhealthy images and finds the difference between the two. These are then used to highlight local and global features from the image which otherwise might have been overlooked. The work in^2^learns a map generating function from the training data. This function then generates an instance specific visual attribution map highlighting the features unique for a class. The VANT-GAN^20^ approach generates VA maps directly from unhealthy images, which can then be used to generate healthy-looking images from unhealthy images. (This latter anticipates that the direct map modelling learns *why* the image is unhealthy and captures the appropriate local and global visual attributes of the disease).

Charachon^40^ generates a range of adversarial examples and tracks the gradient across the stable generation of the original image and the adversarial example. By mapping these gradients to image space, visual attribution maps are generated to find differences between the counterfactuals and the original image.

### Generation of complete counterfactuals

The second (more common) class of generative visual attribution works generate complete subject/image counterfactuals, which are used for diagnostic findings and may or may not be used for explicit subtraction of images for highlighting the differences between the normal and generated counterfactual. STEEX^41^uses region-based selection of images and counterfactuals are generated only using semantic guidance. The regions are thus hoped to be meaningful (such as selecting a traffic signal with a green light and generating a counterfactual for a stop light within a complex image of a traffic junction). The counterfactuals are generated using semantic synthesis GAN, and the generation is constrained to keep the other regions unchanged. The Singla^14^ approach is a similar approach which uses perturbations in the original image controlled by a parameter. A counterfactual is generated for the perturbation such that the posterior probability of the image changes to the desired value of the parameter in the interval [0, 1].

Cutting edge methods of image generation, such as diffusion models, have significantly improved the resolution and quality of generated images. These models have been utilized in counterfactual generation techniques for the latter class of techniques such as Diff-SCM^42^, “What is healthy”^21^and other similar techniques^25,43^. Diffusion models based generative VA techniques include^44^, which use noise encoding with reversed sampling and perform guidance using a class label and task-specific network. This combination is then denoised with a sampling scheme to generate a class conditional counterfactual. Unsupervised Medical Image Translation with Adversarial Diffusion Models^26^use a combination of diffusive and non diffusive models in an adversarial setup, to perform nosing and transformation operations with the noised latents of the image to translate between two modalities of MRI scans, using class conditioning, such as transforming a T1 contrast image to T2. Diffusion Models for Medical Anomaly Detection^25^use a weakly supervised setup for generating healthy counterfactuals of brain tumor images. The approach uses the noised latents from the diffusion model of the image and perform classifier guided denoising of the latent to produce a healthy image without a tumor. The What is Healthy^21^? work similarly encodes the image into noised latents, using an unconditional model. The decoding of the latent can be done via class label or unconditionally, to generate a counterfactual of the starting input image. A heatmap of the region containing the lesion is then produced by taking the difference between the reconstructed healthy and starting image. The guidance is performed without a downstream classifier using conditional attention mechanism techniques.

In both of these broad classes of generative VA approach there is noticeable absence of a linguistic, natural language explanation or conditioning mechanism easily with which a domain expert could engage ‘in the loop’ (e.g. communicating with the system in domain specific terminologies via precise relational instructions for counterfactual generation). Such techniques require the use of classifier guidance for conditional descent of gradients mapping between the latent parameter space and the image space (for example, using weakly supervised decoding strategies or hyperparametric perturbation of the image towards a healthy looking counterfactual). Furthermore, such techniques focus on regions of high information density, in most cases leaving the broad structure of the image remain changed. (An example would be a tumor causing exogenous pressure in the brain such that the surrounding tissue is displaced; this structural deformity would not be visually reversed by the above techniques, but rather just the tumor mass removed, and the unhealthy tissue converted into healthy tissue via transformations of pixel level features characteristic of the affected region).

## Diffusion models

Diffusion models are probabilistic models which learn a data distribution by reversing a gradual noising process through sampling. Denoising thus proceeds from an assumed starting point of *x*(*t*), where *x*(*t*) is considered the final noisy version of the input *x* (which, being assumed to be equivalent to pure noise, can be treated as an easily sampled latent space). The model thus learns to denoise *x*(*t*) into progressively less noisy versions $x(t-1), x(t-2)..$until reaching a final version x(0)^24^, representing a sample from the domain distribution. In transforming a (typically uniformly or Gaussian sampled) latent space into an observational domain, the process is thus one of generative machine learning, with the denoiser typically a deep neural network of learned parameter weights. The latest approaches, however, use the reweighted variant of the evidence lower bound, which estimates the gaussian noise added in the sample *x*(*t*), using a parametrized function $\theta (x(t),t)$ rather than a denoised version of input *x*^45^:

1 $$\begin{aligned} L_{D M}=\mathbb {E}_{x, \epsilon \sim \mathscr {N}(0,1), t}\left[ \left\| \epsilon -\epsilon _\theta \left( x_t, t\right) \right\| _2^2\right] \end{aligned}$$

with $\epsilon _\theta \left( x_t, t\right)$ estimated via the diffusion model, such that the objective function is the difference between the predicted (latent parameter instantiation) noise and the actual noise instantiation (*t* is an arbitrary time step uniformly sampled from 1, . . . , T and $E_x$ denotes the expected value over all examples *x* in the dataset).

### Latent diffusion models

To lower computational demands, latent diffusion models first seek to learn an appropriate latent space, one which, when decoded, is perceptually equivalent to the image space (a key assumption of latent diffusion is thus that noise perturbation of image and latent spaces are not intrinsically incompatible with regard to the generative process). Denoting the encoder by *E*, *E* hence learns to map images $x \in Dx$ into a spatial latent code $z = E(x)$. The essential mechanism of latent diffusion is then as indicated previously going forward - i.e. seeking to learn a model to correctly remove noise from an image, though this time in the latent space. The decoder *D* (which is usually a DNN) learns to map the latent codes back to images, such that $D (E(x))$ $p \thickapprox q$ $x$. The objective function for the latent diffusion model now becomes

2 $$\begin{aligned} L_{L D M}:=\mathbb {E}_{\mathscr {E}(x), \epsilon \sim \mathscr {N}(0,1), t}\left[ \left\| \epsilon -\epsilon _\theta \left( z_t, t\right) \right\| _2^2\right] \end{aligned}$$

where *z*(*t*) is the latent noised to time step *t*^45,46^.

### Latent diffusion autoencoders

The autoencoder model follows a training paradigm similar to^47^ in an adversarial setting, such that a patch-based discriminator $D_\psi$ is optimized to discriminate between original images and reconstructions *D*(*E*(*x*)) in combination with a perceptual loss^48^, ensuring the modes learns the global composition of images well, while preserving locally realistic patterns^45^. The full objective function, utilizing a combination of the two losses discussed above to train the autoencoding model (*E*, *D*) is stated as

$$L_{\text{ Autoencoder } }=\min _{\mathscr {E}, \mathscr {D}} \max _\psi \left( L_{r e c}(x, \mathscr {D}(\mathscr {E}(x)))-L_{a d v}(\mathscr {D}(\mathscr {E}(x)))+\log D_\psi (x)+L_{r e g}(x ; \mathscr {E}, \mathscr {D})\right)$$

An image $x \in \mathbb {R}^{H \times W \times 3}$ in the RGB space is encoded via the encoder *E* into spatial latent code *z*, where $z \in \mathbb {R}^{h \times w \times c}$. Crucially, the image *x* is downsampled by the encoder by a factor of $f=H/h = W/w$, reshaping it into $H/f \times W/f \times 4$using a relative downsampling factor f=8^45^.

### Conditioning using a domain-specific encoder

In the following, the noise prediction function $\epsilon _\theta \left( x_t, t\right)$is implemented using a time-conditioned Unet model^49^, which can also be conditioned on class labels, segmentation masks, or outputs of a jointly trained domain specific encoder. Let *y* be the condition input and $T_{(\theta )}$ be a model which maps the condition *y* to an intermediate representation $T_{(\theta )}(y)$which is then mapped to the intermediate layers of the UNet via a cross-attention layer^50^. The objective function for the class-conditional variant of latent diffusion thus becomes:

3 $$\begin{aligned} L_{L D M}:=\mathbb {E}_{\mathscr {E}(x), y, \epsilon \sim \mathscr {N}(0,1), t}\left[ \left\| \epsilon -\epsilon _\theta \left( z_t, t, \tau _\theta (y)\right) \right\| _2^2\right] \end{aligned}$$

### Image priors

In the above, any arbitrary image can be considered an instantiation of the generative latent parameters. Thus, instead of commencing from pure noise (i.e. purely stochastic latent parametric instantiation), the latent diffusion process can instead be initiated from a given image, via application of the appropriate Stochastic Differential Equations (SDEs), as a form of prior conditioning in the image space. The given image (which may or may not be in the training data distribution, but which is presumed to lie within the manifold of natural images), is firstly perturbed with Gaussian noise (’lifting out the image manifold’). This noise is then removed progressively via the learned denoiser, which effectively acts to reproject the guide image back into the manifold of natural images; This may be thought of as a short random walk *within the manifold* of a given metric distance.

More formally, if $x(0) \sim p_0$ is a sample from the data distribution, the forward SDE produces *x*(*t*) for $t \in (0, 1]$ via Gaussian diffusion. Given *x*(0), *x*(*t*) is distributed as:

4 $$\begin{aligned} x(t) = \alpha (t)x(0) + \sigma (t)z, \; \; z \sim N (0, I) \end{aligned}$$

where the magnitude of the noise *z* is defined by the scalar function $\sigma (t): [0, 1] \rightarrow [0, \infty )$. The magnitude of the data *x*(0) is defined by the scalar function $\alpha (t):[0, 1]\rightarrow [0, 1]$. The probability density function of *x*(*t*) as a whole is denoted $p_t$.

The usually considered SDE are of two types. One is Variance Exploding SDE, where $\alpha (t)=1$ for all *t* and $\sigma (1)$ is a large constant, which makes $p_1$ close to $N(0, \sigma ^2(1)I)$. The second type is the Variance Preserving SDE, satisfying $\alpha ^2(t) + \sigma ^2(t) = 1$ for all *t* with $\alpha (t)\rightarrow 0$ as $t\rightarrow 1$, so that $p_1$ equals to *N*(0, 1)^51^.

Image synthesis is then performed via a reverse SDE^52,53^ from the noisy observation of *x*(*t*) in order to recover *x*(0), given knowledge of the noise-perturbed score function $\nabla x \log p_t(x)$. The learned score model as $s_\theta (x(t), t)$, the learning objective for time *t* is:

5 $$\begin{aligned} L_t=\mathbb {E}_{\textbf{x}(0) \sim p_{\text{ data } }, \textbf{z} \sim \mathscr {N}(\textbf{0}, \textbf{I})}\left[ \left\| \sigma _t \varvec{s}_{\varvec{\theta }}(\textbf{x}(t), t)-\textbf{z}\right\| _2^2\right] \end{aligned}$$

with $s_\theta (x(t), t)$ a parametrized score model to approximate $\nabla x \log p_t(x)$; the SDE solution can be approximated with the Euler-Maruyama method^51^. The update rule from $(t + \Delta t)$ to *t* is:

6 $$\begin{aligned} \textbf{x}(t)=\textbf{x}(t+\Delta t)+\left( \sigma ^2(t)-\sigma ^2(t+\Delta t)\right) \varvec{s}_{\varvec{\theta }}(\textbf{x}(t), t)+\sqrt{\sigma ^2(t)-\sigma ^2(t+\Delta t)} \textbf{z} \end{aligned}$$

A selection can be made on a discretization of the time interval from 1 to 0 and after the initialization $x(0) \sim \mathscr {N} (0, \sigma ^2(1)I)$, Equation 4 can be iterated to produce an image *x*(0)^51^.

### Additional control priors

Additional conditioning mechanisms can be introduced to add further control to the generation e.g. ControlNet^54^ adds intermediate layers to the feature maps at each step of the downscaling operation while transitioning from image to latent space. Thus it becomes possible to add a task-specific image-conditioning mechanism to the model:

7 $$\begin{aligned} \left. \mathscr {L}=\mathbb {E}_{\varvec{z}_0, t, \varvec{c}_t, \varvec{c}_{\textrm{f}}, \epsilon \sim \mathscr {N}(0,1)}\left[ \Vert \epsilon -\epsilon _\theta \left( z_t, t, \varvec{c}_t, \varvec{c}_{\textrm{f}}\right) \right) \Vert _2^2\right] \end{aligned}$$

Where given an image ${z}_0$, noised latents ${z}_t$ are produced by progressively adding gaussian noise to the initial image after time steps *t*. Given the time step *t*, text prompts $c_t$, and task specific conditions $c_f$, the model learns a network to predict the added noise $\epsilon _\theta$. Some examples of task-specific image based conditioning include Canny edge maps, Semantic Segmentaion, Sketch-based guidance, and human pose^54^ etc.

The conditioning mechanisms of input text, image priors, depth and segmentation maps can thus be used in combination with each other, complementing or adding to the image generation for further generative control as required on a task-by-task basis.

## Methodology

In the following, we indicate normal medical images by $I^n$ and abnormal images by $I^a$. We make the assumption that $I^n$ and $I^a$ are sampled from distributions $p_n(I)$ and $p_a(I)$ respectively. Additionally, we assume that the differences between an abnormal image and its corresponding normal image (from the same patient) are only the characteristic disease markers or indicators of diagnostically relevant abnormality, and no other structural differences are present. In this setup, given an input abnormal image $I^a$, we wish to produce a visual attribution map $M(I_i^a)$ that contains all the features that differentiate an abnormal image $I_i^a$ from its normal counterfactual $I_i^n$, such that mapping is decomposed $M(I_i^a) = I_i^a - I_i^n$in common with the VANT-GAN^20^ strategy for visual attribution, albeit in a visual diffusion rather than GAN-based context.

**Fig. 1:**
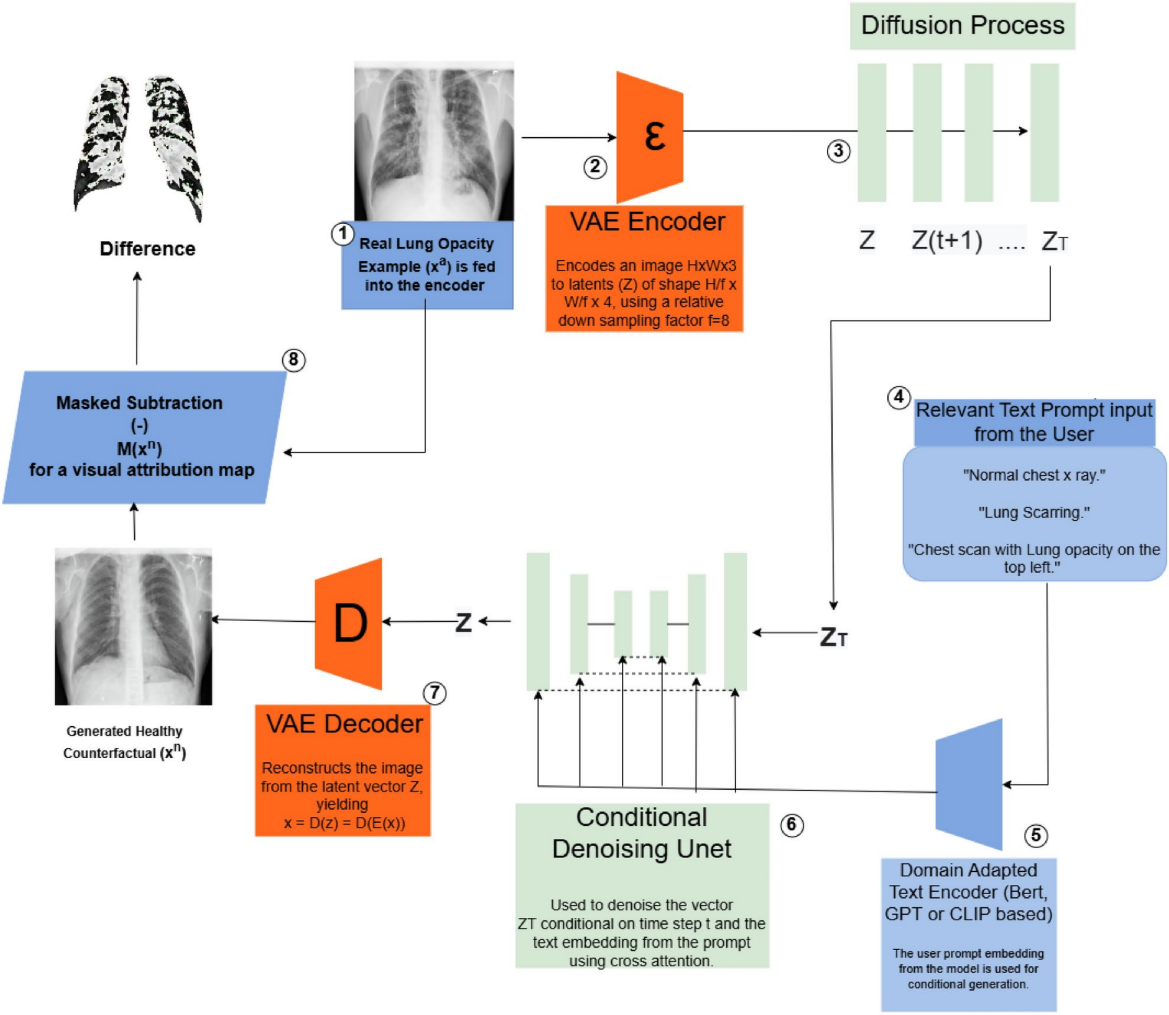
The counterfactual generation pipeline takes as input the abnormal image $x^a$, which is then encoded by the VAE encoder ($\epsilon$) to form the encoded image latents *Z* and passed through the diffusion process to form noised latents of the image $Z_T$ after incremental *t* steps. The fine-tuned conditional U-net denoises the latents into the conditioned latent *Z*, decoded by the VAE decoder *D* into the final generated counterfactual $x^n$, from which a visual attribution map M($x^n$) is subtractively generated.

To generate the normal counterpart $I_i^n$ we use a conditioned stable diffusion model which combines a text and an image condition or input of the forms set out in sections 2.3 and 2.5 via the loss functions delineated in equations 5 and 7. Using an image to image synthesis setting similar to SDEdit^51^, we initiate with the abnormal image as the guide $x^{(g)} = I_i^a$ and add Gaussian noise to form the noised latents $z_t = x^{(g)}(t_0)\sim \mathscr {N}(x^{(g)};\sigma ^2(t_0)I)$ which are then used to produce *x*(0) via application of equation 6, conditioned on $T_\theta (y)$, where $T_\theta$ is a domain adapted text encoder which maps the conditional prompt *y* to an intermediate representation $T_\theta (y)$. Hence the normal corresponding image $I_i^n$ = *x*(0) is synthesized as the denoised version of $\epsilon _\theta (z_t,t,T_{\theta }(y))$. The mask $M(I_i^a)$ is then explicitly produced by subtracting the generated normal counterpart from the abnormal image. The network architecture is depicted in Figure 1.

The conditioned latent diffusion model pipeline that we utilise in the following experiments deploys an initial encoder/decoder network of the form of a variational autoencoder (VAE), a time-conditioned Unet model^49^conditioned on a domain-specific encoder in the textual domain (specifically a Bert based model trained on radiology reports called RadBERT^36^) and, finally, an additional system fine tuning detailed below. We use an image-to-image conditioning mechanism paralleling that of SDEdit^51^, with the model taking two inputs, an image and corresponding text prompt to generate the counterfactual image from which the VA map is derived.

## Experiments

We firstly evaluate counterfactual generation –the generation of healthy counterparts to unhealthy scans– via an investigation of its qualitative impact i.e. the overall *visual plausibility* of the generated counterpart. Following this, we seek to quantitatively analyze the generative perturbation of the tested unhealthy scans in order to determine the utility of the method in its primary mode of VA application. Finally, we explore the latent capacity of the trained system to carry out a series of zero-shot counterfactual generation exercises, in particular: *localized disease induction* and the *induction of diseases from outside the training data* in relation to input healthy scans.

### Training details

The pretrained latent diffusion model *CompVis/stable-diffusionv1-4* and the Bert based model *RadBERT* are obtained from Huggingface https://huggingface.co/StanfordAIMI/RadBERT. These were jointly fine-tuned using a single Quadro RTX 8000 at bf16 precision, with batch size = 2, at a resolution of 512x512px. The models were fine-tuned on the diffusers library using an approach for binding a unique identifier to a specific subject via a class-specific prior preservation loss, Dreambooth^55^, with 1200 training steps used for the Normal class, after which 500 training steps are applied for each of the non-healthy classes, namely Lung Opacity, COVID-19, and Viral Pneumonia, making a total number of training steps of 2700. The greater preponderance of the normal class ameliorates the intrinsic imbalance in dataset, with model convergence inherently slower for the X-ray image domain, being out of the initial distribution. The learning rate was 5e-05 and, for sampling, the PNDM scheduler strength is set at 0.55 with Guidance Scale=4 found to be most effective across all classes for counterfactual generation.

The COVID-19 Radiography Database^56^ contains 10192 normal, 3616 COVID-19, 4945 Lung Opacity and 1345 Viral pneumonia chest x-ray images. The dataset is obtained from https://www.kaggle.com/datasets/tawsifurrahman/covid19-radiography-database. The model is fine-tuned on the images using their respective labels as text prompts i.e *Normal chest scan*, *Lung Opacity*, *Viral Pneumonia*, and *COVID 19*.

**Fig. 2:**
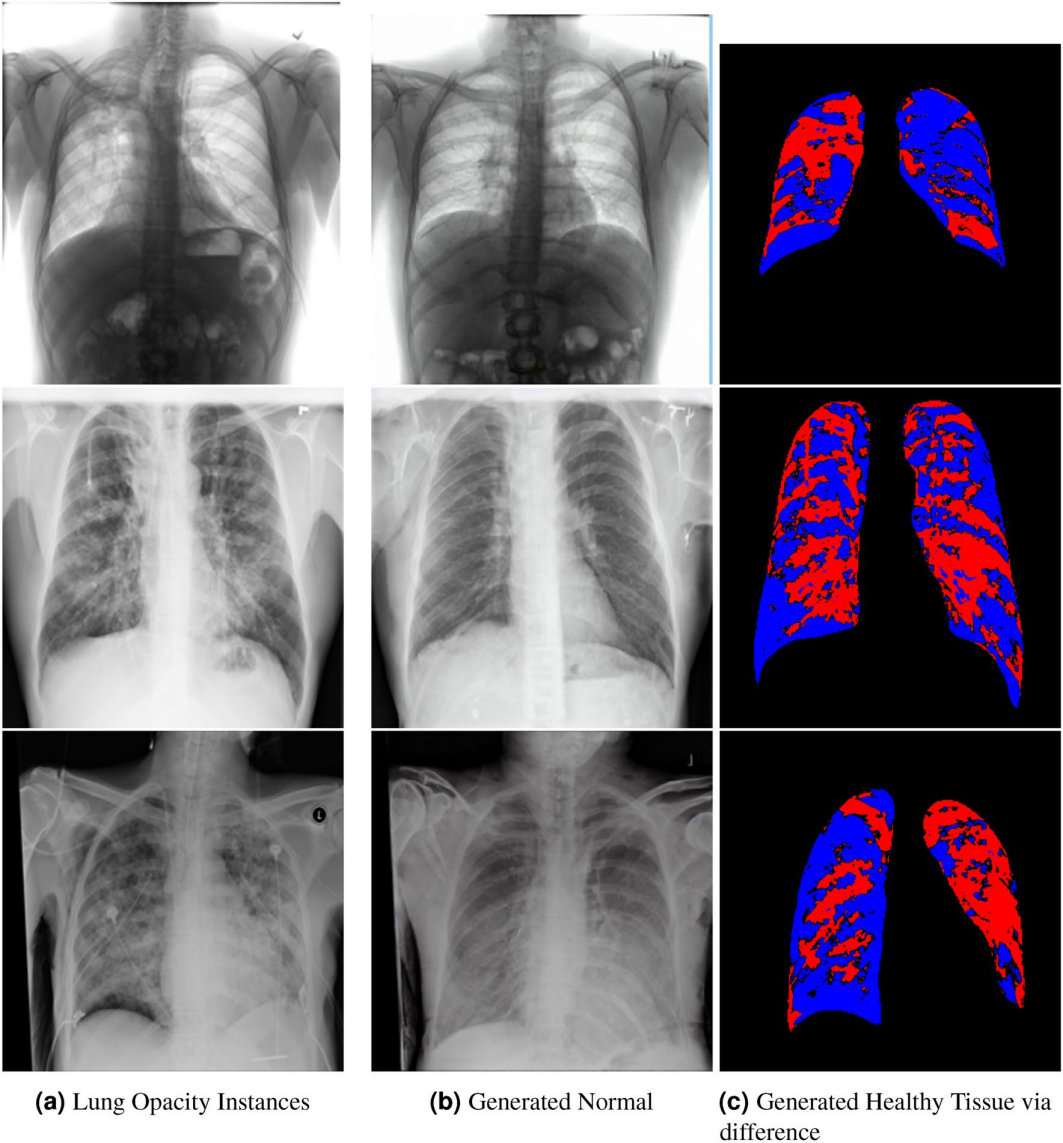
Healthy Counterfactual Generation for three cases of lung opacity (Red indicates generated tissue by the model).

### Qualitative evaluation of healthy counterpart generation

Example images from the disease COVID-19 Radiography Database and their generative healthy counterparts are given in figure 2. The images on the far left are instances of the lung opacity class from the real images in the dataset. The images in the middle column are examples of the generated healthy counterfactuals obtained via latent space diffusion, with RadBERT-guided textual-conditioning via a conditional prompt “normal chest x-ray”. A total of 75 diffusion inference steps are used with image conditioning strength=0.85 and guidance scale=7.5. (The former indicates the level of constraint on changes to the original input image and the latter is the weight given to the textual encoder conditioning in the generation of the image, ranging over [0,1] and [0,9], respectively).

Side-by-side inspection of the generated healthy counterfactuals (as per fig. 2) suggests that, as required, only minimal perturbation is made to the original image with respect to healthy pixels -i.e. localized image sites without structural medical defects. (In the top row, the medical structural defect in the original image is due to a lung opacity, and characterized via a relatively complex interaction between the imaging modality and subject manifesting as ‘gaps’ in the corresponding portions of the lung scan). The healthy/non-healthy discrepancy maps in all of these cases are obtained via masked subtraction of the original image from the generated image (the ground truth segmentation masks correspond to the broad area of interest -i.e. the complete lung). The generated healthy tissue is thus a subset of the mask and is shown in the final column of fig. 2 for the respective cases.

**Fig. 3:**
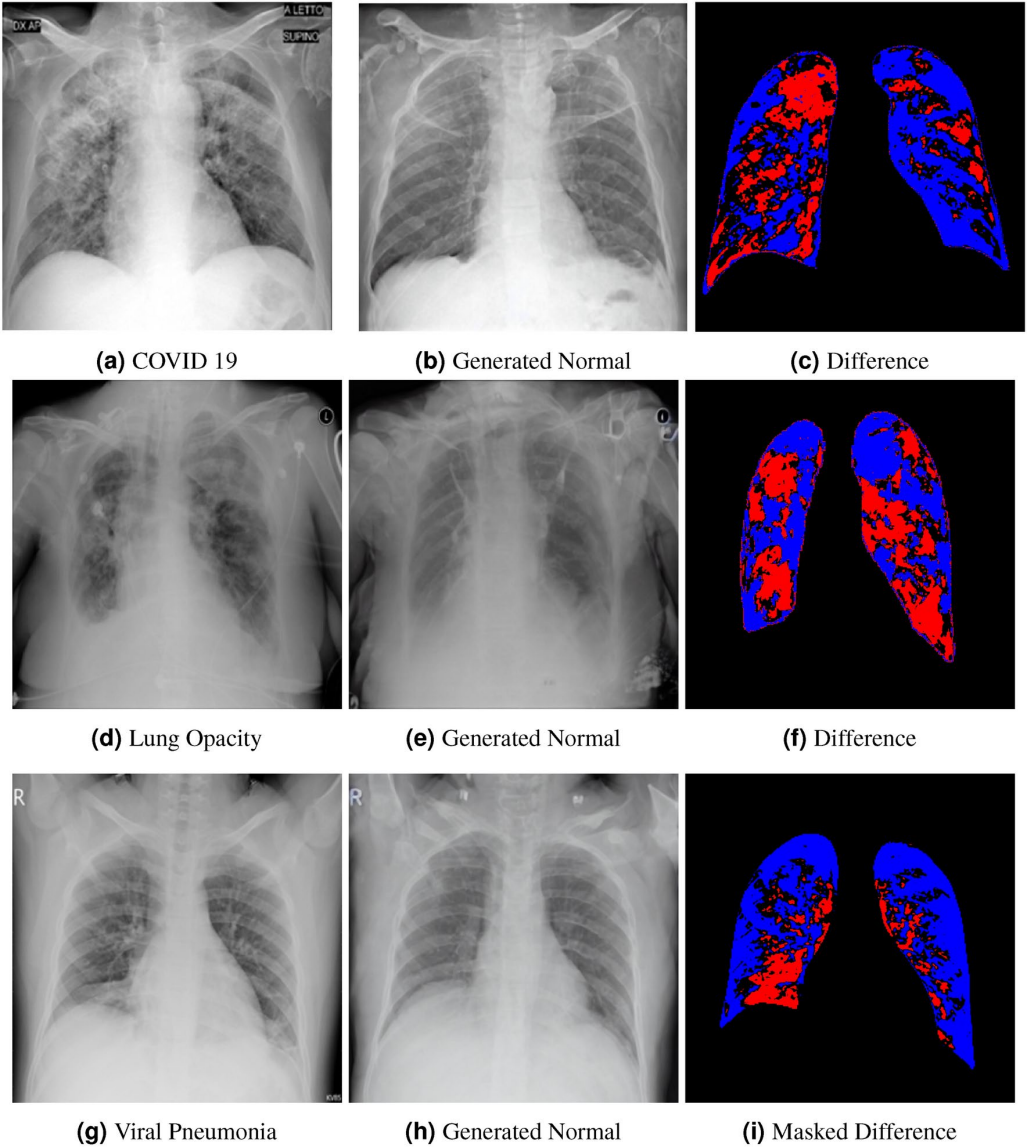
Healthy Counterfactual Generation (Red indicates generated tissue by the model).

In the context of a VANT-GAN^20^-based approach, this highlighted material constitutes the diagnostic counterfactual visual attribution, i.e. the selection of material relevant to the diagnosis of the unhealthy condition. Healthy counterfactual generation was performed for the complete datasets in the three unhealthy classes, i.e *Lung opacity*, *Viral Pneumonia* and *COVID*, examples of which are given in fig. 3 for the three classes (all of the generated healthy counterfactuals from this experiment can be found on https://huggingface.co/ammaradeel/diffusionVA). Visual inspection indicates that the generated counterfactuals are, in general, visually plausible with minimal perturbation made to the unhealthy image overall. Moreover, the healthy counterpart generation does not appear to unnecessarily affect aspects of the images unrelated to the medical condition, the model selectively making changes to the unhealthy regions in a structurally plausible manner, e.g. generating missing portions of the lung without generating extraneous lung material where it would be expected to normally exist (e.g. in the abdominal cavity).

### Quantitative evaluation of healthy counterpart generation

#### Fréchet Inception Distance (FID) measures

For quantitative evaluation on the COVID19 dataset, Fréchet Inception Distance (FID)^57^ was calculated for the generated healthy counterfactuals for each class in order to measure the general level of plausibility, and also to assess how distant the generated counterpart normal distribution is from that of the healthy and diseased image sets.

**Table 1:** FID as a measure of minimum valid perturbations across classes to generate healthy counterfactuals.

Image Set 1	ImageSet 2	Frechet Inception Distance
Lung Opacity	Generated Healthy	27.8
Lung Opacity	Real Healthy	46.9
	**Relative Absolute Difference**	**19.1**
Viral Pneumonia	Generated Healthy	37.63
Viral Pneumonia	Real Healthy	97.6
	**Relative Absolute Difference**	**59.97**
COVID 19	Generated Healthy	32.2
COVID 19	Real Healthy	38.2
	**Relative Absolute Difference**	**6.0**

FID scores are calculated with default characterisations i.e activations of the pool3 layer of the InceptionV3 model with 2048 dimensions (the particular implementation deployed is sourced from the Pytorch FID package^58^). A lower FID would indicate that distribution of the two image sets are similar. Obtained results (cf Table 1) indicate that the real healthy and the generated healthy counterfactuals have relatively similar distributions, with the exception of the Viral Pneumonia class, which has a significantly larger absolute relative difference in FID scores. (An “ImageSet” here indicates randomly-sampled images of a real class or a generated class. E.g. In the first row of Table 1, ImageSet 1 is Lung Opacity, referring to all images of the Lung Opacity class from the original dataset, while ImageSet 2 contains all *generated* healthy images corresponding to ImageSet1. ImageSet 1 and ImageSet2 in the second row correspond to the images of the Lung Opacity and Healthy classes of the *original* dataset respectively).

Relative differences between generated healthy and real healthy images are presented in Table 2 for respective classes (with FID measured as $\left\| \mu _h - \mu _g \right\| _2^2 + \text {Tr}(\Sigma _h + \Sigma _g - 2(\Sigma _h\Sigma _g)^{1/2})$ for the two continuous multivariate Gaussian distributions parametrised $(\mu _g,\Sigma _g)$ and $(\mu _h,\Sigma _g)$ applied to activations of the pool3 layer of the InceptionV3 model).

The relative differences highlighted in Table 2are overall indicative of good fidelity (By way of baseline, FID differences using unconditioned stable diffusion without any training or fine-tuning can reach values 275.0 in the Roentgen^38^ study).

**Table 2:** FID as a measure of image quality.

Image Set 1	ImageSet 2	Frechet Inception Distance
Real Healthy	Generated Healthy from the Lung Opacity class	60.60
Real Healthy	Generated Healthy from the Viral Pneumonia class	110.72
Real Healthy	Generated Healthy from the Viral COVID19 class	45.11

The overall visual soundness of the generated images, as validated via the absolute and relative FID scores obtained for each of the classes, is thus broadly consistent with the previous qualitative interpretation that tested image distributions are minimally perturbed in order to transform them into healthy counterfactuals, while refraining from making changes to the healthy local regions of the image (the scores of the COVID19 class are the closest in this respect among the tested disease conditions, with a relative absolute difference of **6.0** in FID scores between real and generated images.

The scores for the viral pneumonia class appear to be in a large part attributable to the relatively larger magnitude of fundamental structural differences between healthy and viral pneumonia images in the training set: in particular, the viral pneumonia image set mostly had scans from children and infants, while the healthy class was of adult majority. (This data bias would break the basic assumption that differences between class image sets is due only to structural defects of disease).

#### SSIM and MS-SSIM measures

As a further quantitative measure of the relationship between diseased image and generated healthy counterfactuals, we adopt the Structural Similarity (SSIM) and Multi Scale Structural Similarity Metric (MS-SSIM)^59^ metrics, calculated between the unhealthy images and their respective generated counterparts, and averaged across classes.

The Structural Similarity index^60^ quantifies the differences between a processed/distorted image *x* and a reference image *y*, combining the three key comparisons: luminance *l*(*x*, *y*), contrast *c*(*x*, *y*) and structure *s*(*x*, *y*). The SSIM(x,y) between two signals or images *x* and *y* is then given as: $\operatorname {SSIM}(\textbf{x}, \textbf{y})=[l(\textbf{x}, \textbf{y})]^\alpha \cdot [c(\textbf{x}, \textbf{y})]^\beta \cdot [s(\textbf{x}, \textbf{y})]^\gamma$, where $\alpha$, $\beta$ and $\gamma$ are weighting variables, used to control the relative importance of the three factors. We use the general form of the measure where $\alpha = \beta = \gamma = 1$ and $C_3 = C_2/2$:

8 $$\begin{aligned} \operatorname {SSIM}(\textbf{x}, \textbf{y})=\frac{\left( 2 \mu _x \mu _y+C_1\right) \left( 2 \sigma _{x y}+C_2\right) }{\left( \mu _x^2+\mu _y^2+C_1\right) \left( \sigma _x^2+\sigma _y^2+C_2\right) } \end{aligned}$$

with mean intensities $\mu$ and standard deviations $\sigma$, estimating the signal contrast. $\sigma _{x y}$ denotes the covariance of *x* and *y*, while $\sigma _x^2$ and $\sigma _y^2$ denote the variance of *x* and *y* respectively. $C_1$, $C_2$ and $C_3$are constants or combinations of constants to avoid instability based on the dynamic range of pixels^60^.

The Multi-Scale Structural Similarity^61^ (MS-SSIM) is an extension of SSIM incorporating image details at differing resolutions, progressively downsampling *x* and *y* signals using a low-pass filter in factors of 2. The *j*-th contrast and structure comparisons are respectively denoted as $c_j(x,y)$ and $s_j(x,y)$ (the luminance comparison Eq.12 is made at only the largest scale (i.e. original size) at scale *M*. The Multiscale SSIM is then defined:

9 $$\begin{aligned} \operatorname {MS-SSIM}(\textbf{x}, \textbf{y})=\left[ l_M(\textbf{x}, \textbf{y})\right] ^{\alpha _M} \cdot \prod _{j=1}^M\left[ c_j(\textbf{x}, \textbf{y})\right] ^{\beta _j}\left[ s_j(\textbf{x}, \textbf{y})\right] ^{\gamma _j} \end{aligned}$$

MS-SSIM and SSIM metric values are interpreted as measuring the extent of structural similarity between the generated counterfactuals and unhealthy real images: *a priori*, the structure of unhealthy images should not change significantly overall in terms of their broad morphology, but only the requisite minimal perturbations should be made. A low structural similarity indicates larger perturbations to the unhealthy image, and a higher structural similarity indicates smaller overall perturbation: in the extreme cases, 0 would indicate no structural similarity, and 1 would indicate identity of the images. The SSIM and the MS-SSIM measures for the respective disease classes are as depicted in Table 3, and appear consistent with this prior assumption, with only small variation between tested disease classes.

**Table 3:** MS-SSIM and SSIM as a measure of minimum valid perturbations across classes to generate healthy counterfactuals.

Image Set 1	Image Set 2	MS-SSIM	SSIM
COVID	Generated Healthy	0.830	0.798
Lung Opacity	Generated Healthy	0.813	0.780
Viral Pneumonia	Generated Healthy	0.802	0.768

### Latent capacity of the model for open-ended visual analysis

The implicit coupling of a Language Model (LM) with a stochastic image parameterization model embodied by our approach raises the question of whether other use cases are made possible within a VA context, closer to the goal of arbitrary open-ended counterfactual querying of medical data (e.g. in which a medical practitioner might, as part of the diagnostic chain of evidence, ask: “What would this scan look like if the patient were *X* years older and suffered from condition *Y*?”). Thus we seek to establish the presence of *Latent Capabilities* within the model: i.e. capabilities not explicit instilled at training time.

We conduct two sets of (qualitative and quantitative) experiments to evaluate this latent capacity, namely: *Zero-shot Induction of Non-Healthy Counterparts* and *Localized Disease Induction*.

**Fig. 4:**
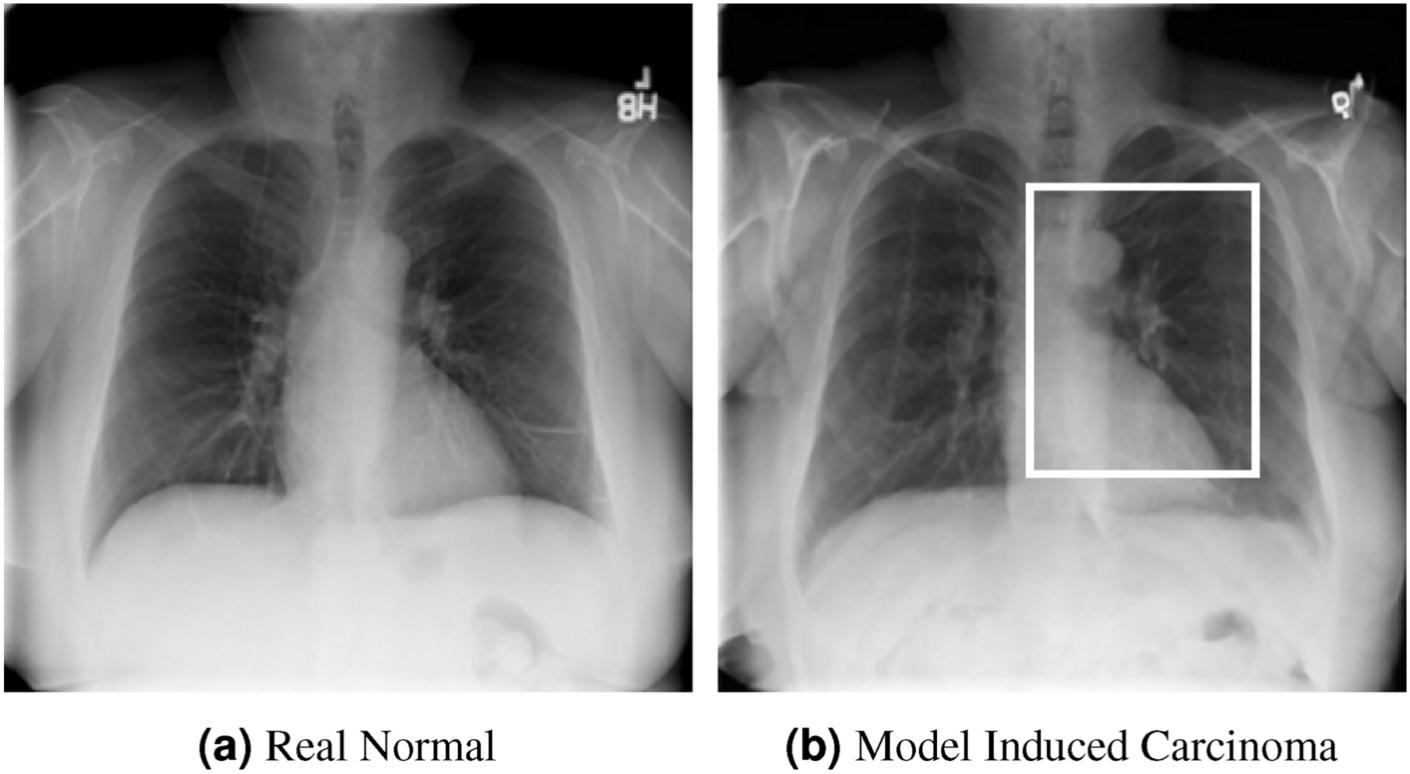
Zero shot carcinoma induction.

#### Zero-shot induction of *non-healthy* counterparts

Despite our model being trained for healthy counterpart generation, we may consider instead a *reverse* of this process, i.e. the induction of a specific disease within healthy scans using the same experimental pipeline. In particular, we can consider the capacity to *induce* disease via the latent language capacity of the model.

As an instance of this, the trained model was prompted in the generative setting for “carcinoma” in relation to a healthy image. The result is shown in Figure 4alongside the real healthy scan and a separate real-case carcinoma can be observed in the image^62^. It is clear that the induced disease is visually comparable to that of the real case despite it’s absence from the training set. We propose that this capability arises as a result of a the internal correlation of the domain-adapted text encoder to that of the visual domain via the visual model, given that the domain-adapted text encoder is trained on the full panoply of Radiology reports.

To evaluate this in more detail we examine a less localised condition: *Cardiomegaly*.

**Zero-shot evaluation: Cardiomegaly**

The disease cardiomegaly (enlargement of the heart) was not present in the training data; to evaluate zero shot induction in this context, we take real images from the small version of the Chexpert^63^ dataset (from https://www.kaggle.com/datasets/ashery/chexpert). Thus, 8060 images of positively identified cases of cardiomegaly were used as the reference image set for real cardiomegaly. Correspondingly, for each of the healthy images from the COVID 19 database, an induced version was generated by the model with the prompt “Cardiomegaly”. FID scores between the real cases of cardiomegaly from the Chexpert dataset and the generated images are given in Table 4.

**Fig. 5:**
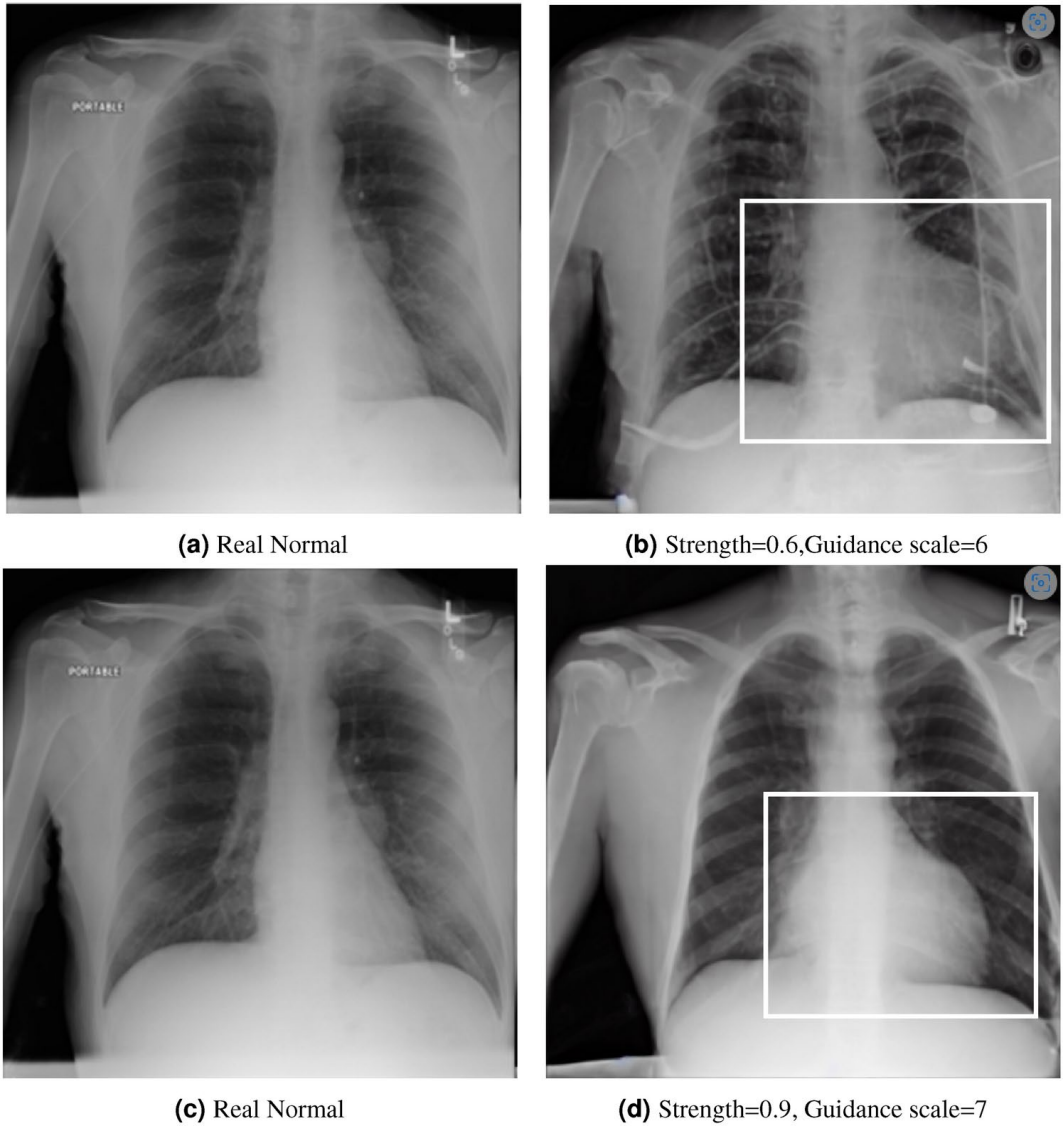
Induction of Cardiomegaly in real healthy scans.

**Table 4:** FID as a measure of minimum valid perturbations for zero-shot cardiomegaly induction.

Image Set 1	Image Set 2	FID
Real Cardiomegaly	Generated Cardiomegaly	52.08
Real Healthy	Generated Cardiomegaly	17.71

The FID scores in Table 4indicate that the generated cardiomegaly images do not have a large distance (using the 275.0 baseline of the Roentgen^38^ study) from the real images from which they were generated, suggesting appropriate perturbations were made and the generations were reasonably close to the real cardiomegaly set from the Chexpert dataset.

Interestingly, while generation across different settings of the visual diffusion hyperparameters *Strength* & *Guidance-scale* did not have a very significant difference on FID scores evaluated across the full range of image sets, visual differences for individual images could be more significant, as highlighted in Figure 5 for two different settings of the respective hyperparameters. This is presumably due to the different aspects specific to individual patient image (such as the prior health of the patient, structural variances due to age, recording equipment, size etc) acting to mimic hyperparametric variation, which primarily appears to affect the opacity of the induced material for hyperparameter settings ranges consistent with good image generation (in general, the *Strength* hyperparameter give scope for larger perturbation from the original image during diffusion, while *Guidance-scale* determines the intensity of text prompt conditioning; optimal settings of these parameters are inherently disease-specific given the wide variation in the amount of pixel opacity needing to be added in the disease induction setting of the pipeline).

**Fig. 6:**
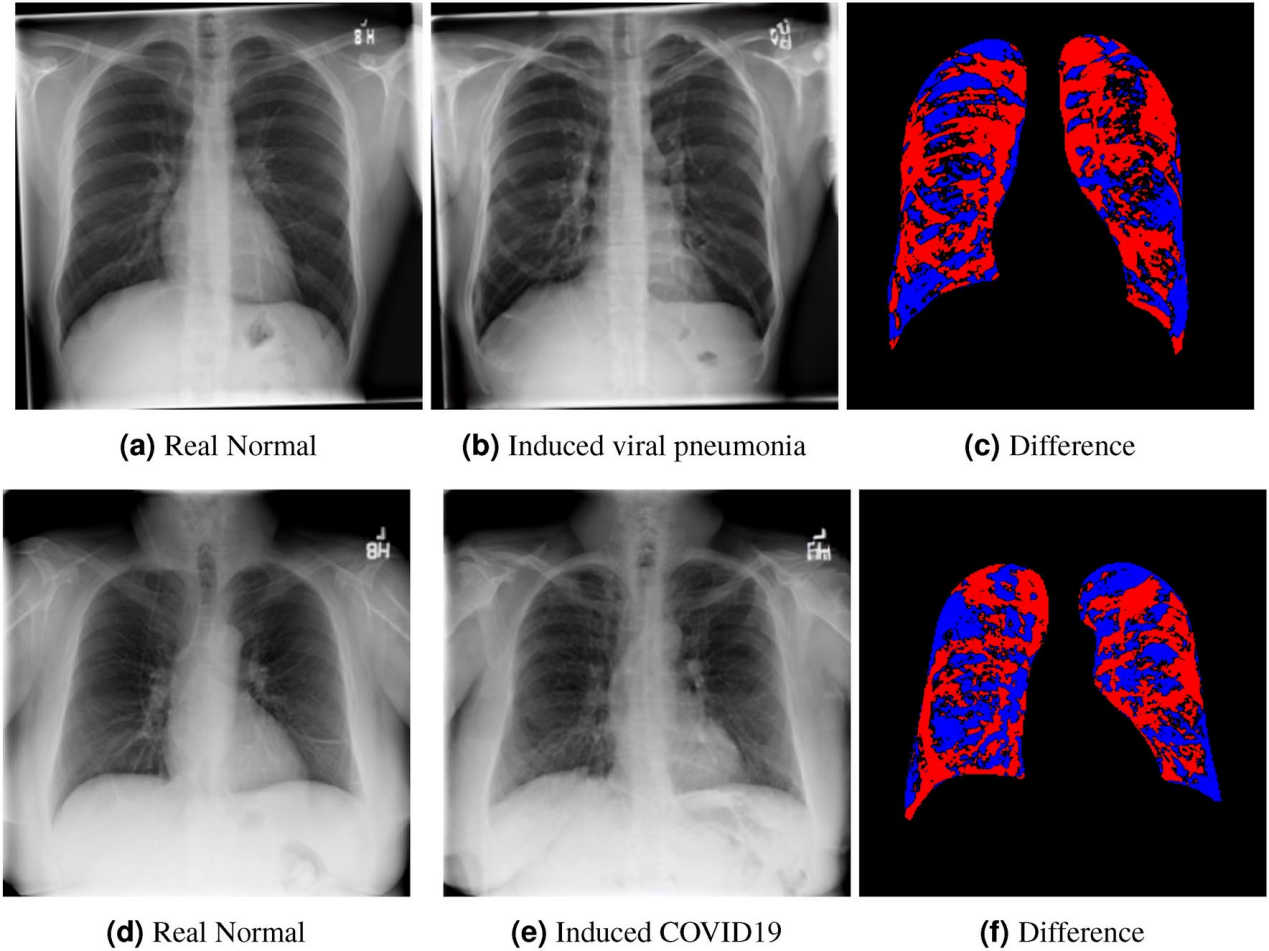
Induction of baseline diseases in real healthy scans (Red indicates induced scarring).

(For an additional comparison baseline, we include results for induction of disease that are within the training set, namely viral pneumonia and COVID19. Results are given in figure 6).

#### Localized disease induction

Finally, a key requirement of counterfactual visual attribution is sensitivity to both *exogenous* and *endogenous* aspects of disease: we define the endogenous visual aspects of disease as those attributes intrinsic to diagnosis, and the exogenous aspects as free-parameters associated with diseased tissue that are not themselves directly implicated in diagnosis. An example might be a tumor identified via its texture characteristics (endogenous), but which is otherwise located arbitrarily within a particular organ (so that *location within the diseased organ* is effectively an exogenous free variable within a VA context).

We therefore illustratively test our model in regard to its latent capability to induce disease in specific locations through the simple expedient of conditioning on positionally-indicative text. The results may be seen in figure 10 for the case of localized lung opacity (lung opacity being chosen because it is both diffuse and generally specific to one or other lung). The respective condition texts are “large lung opacity on the left” and “large lung opacity on the right”.

### Hyperparametric ablation studies

The impact of the hyperparameters *Strength* and *Guidance Scale* were previously indicated in the context of the spectrum of disease severity in *Cardiomegaly*. We here seek to perform a proxy *ablation study* by isolating the individual effects of the respective components of the generative pipeline through the setting of these hyperparameters. In individual terms, the respective hyperparameters act in the following manner:

**Strength:** The Strength parameter dictates the resemblance of the generated image to an original image given as prior conditioning. In contrast to the standard diffusion process, in which the starting point *x*(*t*) can be characterised as pure noise, an image prior is instead used. Noise from some suitable function $\alpha (t)$ (with range [0, 1]) is thus added to an image prior obtained externally or sampled from the data distribution with a multiplicative magnitude coefficient characterised by the Strength parameter. Hence the lower the Strength hyperparameter, the lower the level of additive noise in the image, giving rise to a denoised final image resembling (i.e. proximal in the image subspace to) the image prior *x*(0). A Strength value close to 1, by contrast, introduces large noise levels, resulting in a final denoised image that is relatively dissimilar to the image prior (though still within the image manifold). In the following, this also impacts the inference time, as more additive noise requires an increased number of denoising steps to produce a convergent final image: inference time on the indicated hardware ranges between 1 and 5 seconds, more-or-less in proportion to the *Strength* setting.

**Guidance Scale:** The Guidance Scale parameter controls the effect of the domain-specific encoder on the conditional generation of the image (details of which are discussed in section 2.3); in this case specifically the domain-adapted text encoder. The *Guidance Scale* has a range of [0, 9] indicating the degree of alignment of the generated image with the prompt (i.e. the textual prior conditioning). An amount close to 0 indicates the highest level of deviation allowance from the prompt.

Critically, since these two parameters control the degree of influence of the key *pipeline components*, in particular those of the text encoder and the image prior, we can use these parameters to conduct a proxy ablation study, eliminating individual components from the pipeline to assess the effect on image quality.

#### Hyperparametric elimination of image priors

In this initial ablation experiment we attempt to generate healthy images conditioned on the text encoder, commencing from pure noise. Eliminating image priors by setting the *Strength* parameter to 0.99 and the *Guidance Scale* to 8.5 we generate 4000 images with the prompt ”healthy chest scan”. The generated set is then compared to the real healthy set for image quality. The FID from the real healthy for the respective generated image sets are presented in table 5.

**Fig. 7:**
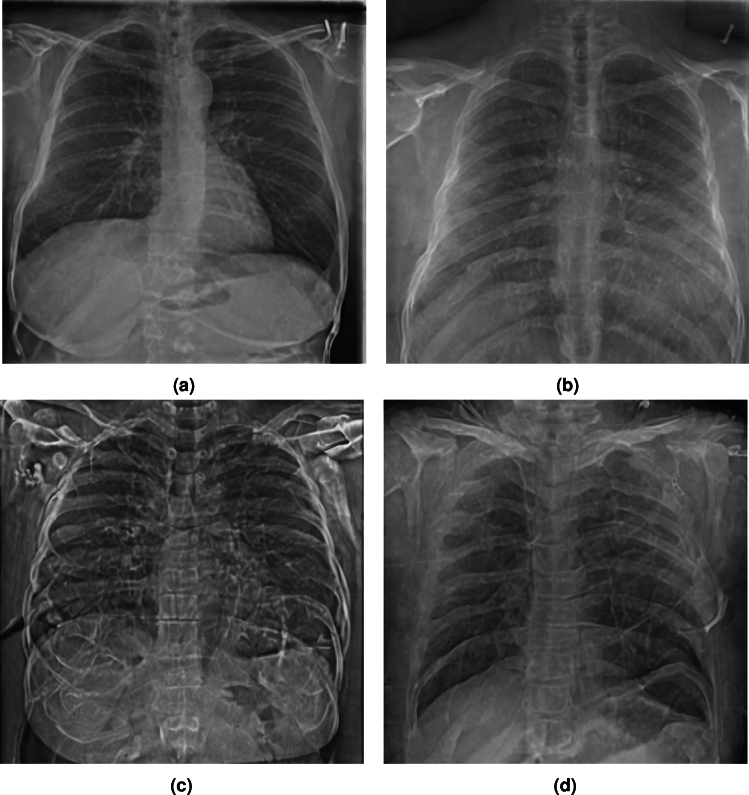
Example Images: Hyperparametric Elimination of Image Priors using the prompt “healthy chest scan”.

**Table 5:** FID as a measure of component effect on overall image generation.

Image Set 1	Image Set 2	FID
Real Healthy	Generated eliminating Text Encoder	91.57
Real Healthy	Generated eliminating Image Priors	113.14
Real Healthy	Generated eliminating Text Encoder and Image Priors	113.71

Under these settings, the generated images exhibit a lack of overall structure and quality. As illustrated in 7b the generations have relatively poor quality: in fact, the the model often fails to generate the basic structure of a chest scan (Images excluded as they might be disturbing). Image priors are thus crucially helpful in controlling the VA generation process as the modality of the output is effectively that of input (i.e. Image to image). Starting from a base anatomical structure, adding details according to a condition from the prompt significantly assists induction. Example generations are presented in Figure 7.

#### Hyperparametric elimination of the text encoder

To observe the effect of the domain adapted text encoder, we eliminate it from the generation pipeline using the *Guidance Scale* parameter with a value of 0.01 - and the prompt as an empty string. We start with a random healthy image and set the *Strength* to 0.4 to introduce a degree of noise in the input image to give a degree of diversity in the generations. 4000 images are generated with the aforementioned hyperparameter settings for comparison with the real healthy set of chest scan images.

**Fig. 8:**
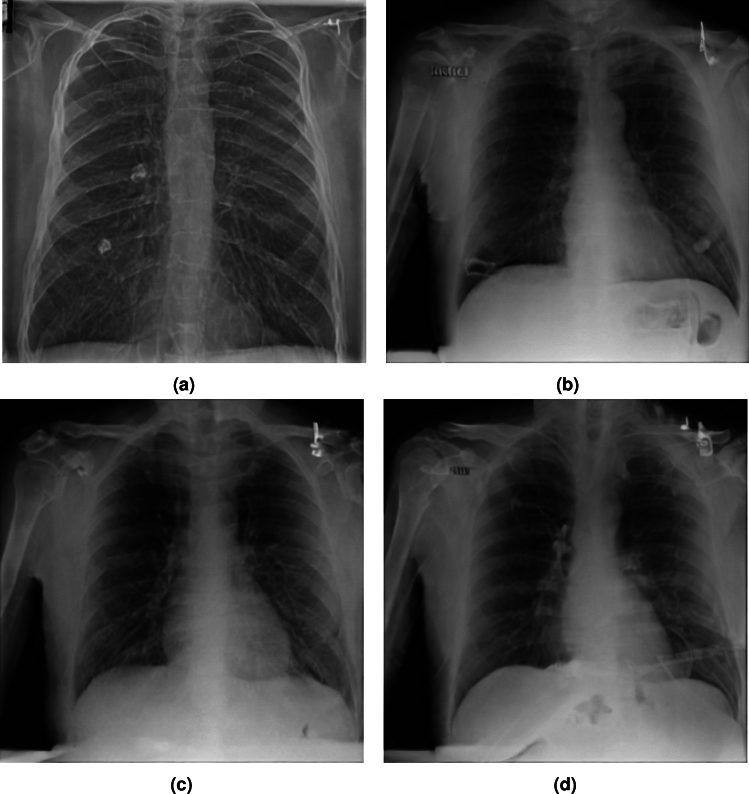
Elimination of the Text Encoder, with a healthy scan as image prior.

The images in Figure. 8 indicate that the generation is of a relatively better quality compared to images generated without image priors, with a FID from the original healthy set of 91.57. Generations remain consistently close to the original image prior in overall structure, and only very slightly deviate in terms of lung mass (A different setting of *Strength* would introduce a greater degree of diversity amongst the generations in terms of overall structure, but was avoided in order to isolate effects of the text encoder).

####  Unconditional image generation - hyperparametric eliminating the text encoder *and* image priors

**Fig. 9:**
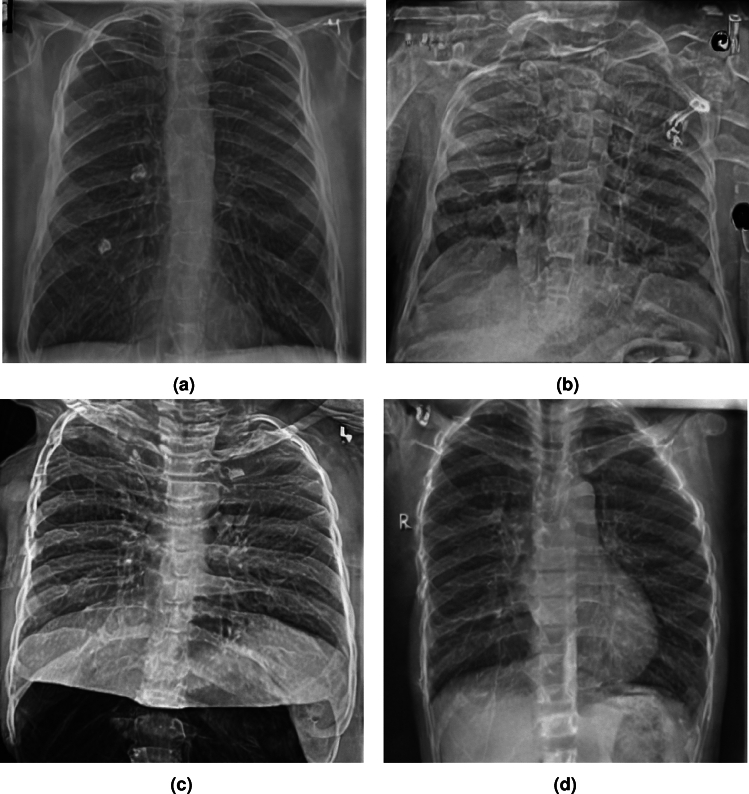
Elimination of the Text Encoder and Image Priors.

In the final phase of the ablation study, we eliminate the text encoder *and* the image priors to give completely unconditional image generation. Commencing with pure noise, setting the *Strength* to 0.99, *Guidance scale* to 0.1, and an empty string as the prompt, 4000 images are generated. With an average FID of 113.71 this setting results, as anticipated, in the greatest distance to the healthy set in terms of image quality. The images of Figure. 9 exhibit relatively greater diversity than the other settings, and visually have the largest amount of structural inconsistency in relation to generation of normal chest scans. The FID value is relatively similar to the setting in which only the text encoder is used as a condition on generation, though the visual results would suggest a far poorer result.

We conclude, finally, that all components of the pipeline are critical in the visual attribution approach. Although beyond the scope of the current ablative analysis, further light may be shed on the impact of individual components via the use of alternative metrics such as conditional FID, domain adapted models for calculating FID, directional difference metrics, or classifier based metrics such as Verisimilitude^19^.

**Fig. 10:**
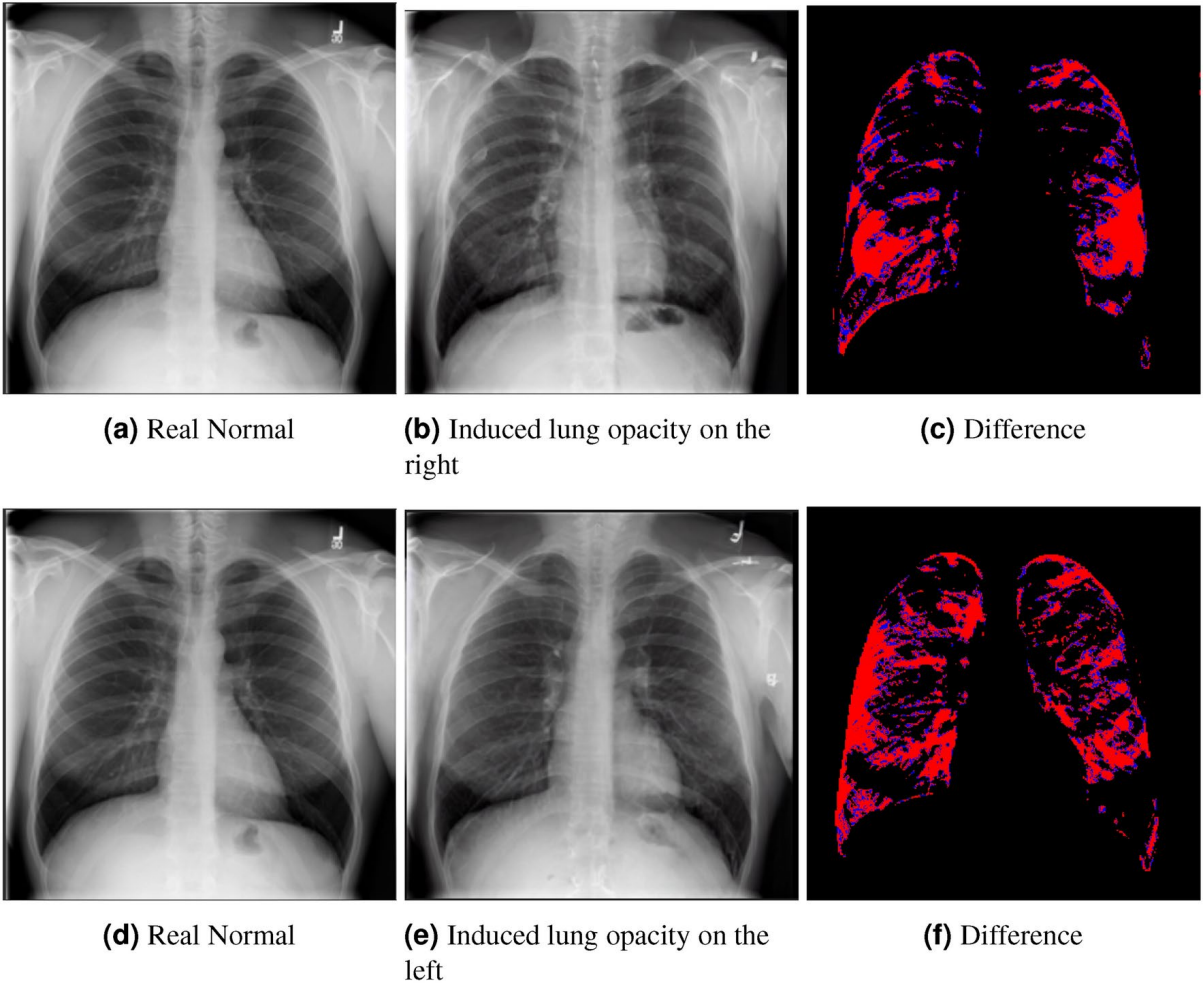
Localized lung opacity induction in healthy scans.

## Conclusion

In this work, we present a novel generative visual attribution technique for improving explainability in the medical imaging domain, leveraging a fusion of vision and large language models via the stable diffusion pipeline, built on foundational generative VA concepts from the VANT-GAN^20^ approach. The model developed generates normal counterparts of scans affected by different medical conditions in order to provide a subtractive salience map between the real affected regions and the generated normal scans, thereby providing insight into those *regions relative to diagnosis* (and which is thus distinct from straightforward segmentation of diseased regions typically associated with machine medical diagnostics). It does so in a manner potentially synonymous with, and therefore assistive to, the inference process of human medical practitioners.

The pre-trained domain-adapted text and vision encoder are jointly fine-tuned using a modest number of image and one-word text training examples from the medical imaging domain for image-to-image generations. The generation capabilities include the induction of different medical conditions in healthy examples induced with varying severity. Inputs to the text encoder support advanced medical domain language and terminology, with the capacity for specifying particular topological locations in organs. Ablation studies highlight the individual and combined contribution of the text encoder and image priors to the generation pipeline.By harnessing the model’s learned multimodal knowledge from the domain-adapted text encoder and the vision model, out-of-training data distribution or zero-shot generations can be made for unseen medical conditions. Similar to other generative architectures, diffusion models have limitations, including but not limited to hallucinations, mode interpolation and memorization. In the medical diagnostics domain, future work will address the possibility of addressing complex disease-interactions, for example, providing simulation of the composite effects of age, lifestyle choices, and differing underlying disease conditions. The modest data requirement may also prove helpful for few-shot learning in relation to rare diseases or those with limited examples (for example, neonatal medical scans).

## Data Availability

The datasets generated and/or analysed during the current study are available in the COVID-19 Radiography Database^56^, CheXpert-v1.0-small^63^, and diffusionVA repositories https://www.kaggle.com/datasets/tawsifurrahman/covid19-radiography-database, https://www.kaggle.com/datasets/ashery/chexpert, and https://huggingface.co/ammaradeel/diffusionVA respectively.
